# Visualization of spatiotemporal dynamics of human glioma stem cell invasion

**DOI:** 10.1186/s13041-019-0462-3

**Published:** 2019-05-06

**Authors:** Ryota Tamura, Hiroyuki Miyoshi, Oltea Sampetrean, Munehisa Shinozaki, Yukina Morimoto, Chizuru Iwasawa, Raita Fukaya, Yutaka Mine, Hirotaka Masuda, Tetsuo Maruyama, Minoru Narita, Hideyuki Saya, Kazunari Yoshida, Hideyuki Okano, Masahiro Toda

**Affiliations:** 1 Department of Neurosurgery, Keio University School of Medicine, 35 Shinanomachi, Shinjuku-ku, Tokyo, 160-8582 Japan; 2 Department of Physiology, Keio University School of Medicine, 35 Shinanomachi, Shinjuku-ku, Tokyo, 160-8582 Japan; 3 Division of Gene Regulation, Institute for Advanced Medical Research, Keio University School of Medicine, 35 Shinanomachi, Shinjuku-ku, Tokyo, 160-8582 Japan; 4 Department of Obstetrics and Gynecology, Keio University School of Medicine, 35 Shinanomachi, Shinjuku-ku, Tokyo, 160-8582 Japan; 5 Department of Pharmacology, Hoshi University School of Pharmacy and Pharmaceutical Sciences, 2-4-41 Ebara, Shinagawa-ku, Tokyo, 142-8501 Japan; 6 Department of Neurosurgery, Fuji Hospital, 137-1 Nishiyashiki, Chiryu-shi, Aichi 472-0007 Japan

**Keywords:** Glioblastoma, Glioma stem cell, Invasion, Brain slice culture, Time-lapse imaging, Tissue clearing

## Abstract

Glioblastoma exhibits phenotypic and genetic heterogeneity, aggressive invasiveness, therapeutic resistance, and tumor recurrence, which can be explained by the existence of glioma stem cells (GSCs). In this study, we visualized the spatiotemporal dynamics of invasion of human GSCs in an orthotopic xenograft mouse model using time-lapse imaging of organotypic brain slice cultures and three-dimensional imaging of optically cleared whole brains. GSCs implanted in the striatum exhibited directional migration toward axon bundles, perivascular area, and the subventricular zone around the inferior horn of the lateral ventricle. GSCs migrated in a helical pattern around axon bundles in the striatum and invaded broadly in both the rostral and caudal directions. GSCs in the corpus callosum migrated more rapidly and unidirectionally toward the contralateral side with pseudopod extension. These characteristics of GSC invasion shared histological features observed in glioblastoma patients. Spatiotemporal visualization techniques can contribute to the elucidation of the mechanisms underlying GSC invasion that may lead to the development of effective therapy for glioblastoma.

**Electronic supplementary material:**

The online version of this article (10.1186/s13041-019-0462-3) contains supplementary material, which is available to authorized users.

## Introduction

Glioblastoma is the most common and aggressive form of primary brain tumors [1] and exhibits a high degree of phenotypic and genetic heterogeneity [2]. Complete surgical removal of tumor cells is difficult because glioblastoma frequently invades across the corpus callosum resulting in bi-hemispheric lesions that have a “butterfly” appearance. The current standard treatment following surgical resection, which includes radiotherapy and chemotherapy with temozolomide, provides only a modest survival benefit [3].

The presence of glioma stem cells (GSCs) that have self-renewal and tumor-initiating capacity has been demonstrated [4–6]. GSCs are thought to be an underlying cause of therapeutic resistance and tumor recurrence [7–9]. Therefore, molecular markers that identify GSCs may be a key for the development of therapeutic approaches targeting GSCs. While many putative GSC molecular markers, including CD133 and nestin, have been identified, they are inconsistent and cannot universally define GSCs due to remarkably high intertumoral and intratumoral heterogeneity [10].

Xenograft-based animal models of glioblastoma are invaluable for the study of the biology of human glioblastoma in vivo and for the evaluation of therapeutic effects [11]. An orthotopic xenograft mouse model has been widely used with human glioblastoma cell lines, such as U87 and U251. However, non-invasive tumor mass is formed and the tumor margin is clearly defined in these xenografts, which do not display the histopathological features of glioblastoma [12–15]. In contrast, xenografts generated by the transplantation of biopsies or GSCs from patients with glioblastoma recapitulate both the genetic and histological features of the primary tumor [11, 16, 17]. Recently, we have isolated GSCs from human glioblastoma specimens [18]. These established cell lines, including hG008, have the potential of high tumorigenesis and aggressive invasiveness.

Visualization techniques have been developed for brain images at high resolution. The organotypic brain slice culture system, which is ideally suited for temporal analysis, serve as a valuable ex vivo tool for various fields of neuroscience [19–23], although global visualization is difficult. For spatial analysis, tissue-clearing methods enable three-dimensional (3D) imaging of whole brain with cellular resolution [24–27]. Here, we report for the first time the spatiotemporal characterization of human GSC invasion in an orthotopic xenograft mouse model using time-lapse imaging of organotypic brain slice cultures and 3D imaging of optically cleared whole brains.

## Methods

### Cell culture

The human glioblastoma cell line U87 was obtained from the American Type Culture Collection. U87 cells were cultured in DMEM (Sigma-Aldrich, St. Louis, MO, USA) containing 10% fetal bovine serum, 2 mM L-glutamine, 100 units/mL penicillin, and 100 μg/mL streptomycin. The human GSC line hG008 was established from human glioblastoma specimens (Additional file 1) [18]. Written informed consent was obtained from the patient. hG008 cells were cultured in Ultra-Low attachment cell culture flasks (Corning, Kennebunk, ME, USA) in DMEM/Ham’s F-12 with HEPES (Wako, Osaka, Japan) containing 2% B-27 (Thermo Fisher Scientific, Waltham, MA, USA), 20 ng/mL recombinant human fibroblast growth factor-basic (PeproTech, Rocky Hill, NJ, USA), 20 ng/mL recombinant human epidermal growth factor (PeproTech), 1000 units/mL recombinant human leukemia inhibitory factor (Nacalai Tesque, Kyoto, Japan), and 1 unit/mL heparin.

### Lentiviral transduction

U87 cells and hG008 cells were transduced with the lentiviral vector CSII-EF-*ffLuc* containing the *ffLuc* gene (a Venus fluorescent protein [28] and firefly luciferase fusion gene) under the control of human elongation factor 1 α subunit (EF-1α) promoter [29]. Transduced cells were seeded as single cells into a 96-well plate and expanded. Single-cell clones stably expressing *ffLuc* were established.

### Orthotopic xenograft

Female BALB/c nude mice (20 g, 6 weeks old) (Sankyo Labo Service Corporation, Tokyo, Japan) were anesthetized with equithesin and placed in a stereotaxic apparatus (Narishige Scientific Instrument Lab, Tokyo, Japan). U87 cells or hG008 cells (1 × 10^5^ cells in 2 μL of phosphate-buffered saline (PBS)) were implanted in the right striatum using a 10-μL Hamilton syringe to a depth of 3 mm from the brain surface through the burr hole 2 mm lateral to the bregma. U87 cells were also implanted in the right cortical area, subventricular zone, or corpus callosum for organotypic brain slice culture. All experiments were performed in accordance with the Guidelines for the Care and Use of Laboratory Animals of Keio University (Approval number: 14057) and the Guide for the Care and Use of Laboratory Animals (National Institutes of Health (NIH), Bethesda, MD, USA).

Mice were sacrificed and transcardially perfused with 4% paraformaldehyde (PFA) at the indicated time points. Brain tissues were fixed with 4% PFA followed by cryoprotection by soaking in 10 and 20% sucrose at 4 °C overnight. Twenty-μm thick coronal sections were cut with a REM-700 microtome (Yamato Kohki, Saitama, Japan). Sections were stored in sterile antifreeze solution at − 20 °C [30].

### Organotypic brain slice culture and image analysis

At 7 days (U87) or 45 days (hG008) after implantation, brain tissues were obtained without perfusion and were sliced into 200-μm thick sections using a Vibratome (Leica, Wetzlar, Germany). The corticostriatal slices containing U87 cells or hG008 cells were placed on Millicell cell culture insert (PICM0RG50; Merck KGaA, Darmstadt, Germany) and transferred to a 3.5-cm glass-bottom dish with 1.8 mL of culture medium. Time-lapse imaging of slice cultures was performed using a confocal laser scanning microscope FV10 (Olympus, Tokyo, Japan), equipped with a temperature and gas supply control system. Images were captured every 20 min during the 144-h culture period, and the photo-bleaching effect was not observed. Image processing was performed using Xcellence software (Olympus). Other serial slices were fixed with 4% PFA every 12 h for 144 h and embedded into paraffin blocks for mutually synchronized histopathological analysis.

3D cell tracking was performed using Imaris image analysis software (Bitplane, Zurich, Switzerland), and tracks were generated based on the Z-stacks of time-lapse confocal fluorescent images. The cell migration tracks were quantitatively parameterized in terms of several metrics. Migration speed, direction, and distance connecting the start and end of the cell tracks were measured, because the cell migratory behavior was further characterized using those three indices. The length of pseudopod was quantified with ImageJ software (NIH) from 2D projections of the imaged volume.

### In vivo bioluminescence imaging

A Xenogen-IVIS 100 imaging system (PerkinElmer, Waltham, MA, USA) was used for in vivo bioluminescence imaging (BLI). Tumor growth was monitored once per week after implantation. Mice anesthetized with isoflurane gas were intraperitoneally injected with 300 mg/kg D-luciferin (VivoGlo Luciferin; Promega, Madison, WI, USA) and placed on a warmed stage inside the camera box of the IVIS imaging system coupled with cool CCD camera using software v2.5. Images were quantified as photons per second for U87 cells, and per minute for hG008 cells.

### Whole-brain clearing

Mice were perfused with 4% PFA at 45 days after implantation of hG008 cells. For preparation of PASSIVE CLARITY-processed mouse brains, brain tissues were fixed with 4% PFA at 4 °C overnight and then incubated in hydrogel solution (4% PFA, 4% acrylamide, 0.25% VA044 in PBS) at 4 °C for 3 days [24]. Brain tissues were degassed and polymerized in the same hydrogel solution at 37 °C for 3 h. Four-mm thick coronal sections, except cerebellum and olfactory bulb, were cut. Hydrogel-embedded tissue sections were washed with clearing solution (200 mM sodium borate buffer (pH 8.5) containing 4% SDS) at 37 °C with shaking for 2 h. Sections were then incubated in fresh clearing solution at 48 °C for 5 days. Imaging was performed by multi-photon microscopy (FLUOVIEW FVMPE-RS; Olympus) and 3D images were reconstructed using FV31S-SW software with a maximum intensity projection algorithm (Olympus).

### Immunohistochemical analysis

For immunocytochemistry, cells were plated on 12-mm cover slips coated with poly-L-lysine or poly-L-ornithine/fibronectin-coated chamber slides. The cultured cells were fixed with 4% PFA and permeabilized with 0.5% Triton-X. For immunohistochemistry, 3-μm paraffin-embedded tissue sections from slice cultures and 20-μm tissue sections from in vivo experiments were used. Samples were stained with the following primary antibodies (diluted 1:200): anti-NeuN (mouse IgG1; MAB377, Merck KGaA), anti-GFAP (rat IgG2a; 13–0300, Thermo Fisher Scientific), anti-O4 (mouse IgM; MAB345, Merck KGaA), anti-human Nestin (rabbit IgG; 18741, Immuno-Biological Laboratories, Gunma, Japan), anti-human Neurofilament protein (mouse IgG1; IS607, DAKO, Glostrup, Denmark), and anti-mouse CD31 (rat IgG2a; 550274, BD Biosciences, Tokyo, Japan). The primary antibodies were detected using Alexa Fluor 555-, Alexa Fluor 568-, or Alexa Fluor 647-conjugated secondary antibodies (diluted 1:500) (Thermo Fisher Scientific). Samples were mounted with VECTASHIELD Antifade Mounting Medium containing DAPI (Vector Laboratories, Burlingame, CA, USA) and examined by fluorescence microscopy (BZ-9000 Biorevo; Keyence, Osaka, Japan). Histological characteristics were assessed using hematoxylin and eosin (H&E) staining.

### Statistical analysis

Student’s *t*-test was used to compare the speed and distance of cell migration between U87 and hG008 cells. One-way ANOVA followed by post-hoc test was used to compare the speed and direction of cell migration and the length of pseudopod in different brain areas. Statistical analyses were performed with IBM SPSS statistics (IBM, Armonk, NY, USA). A *P*-value of < 0.05 was considered statistically significant.

## Results

### In vitro and in vivo growth characteristics of U87 cells and hG008 cells

**Fig. 1:**
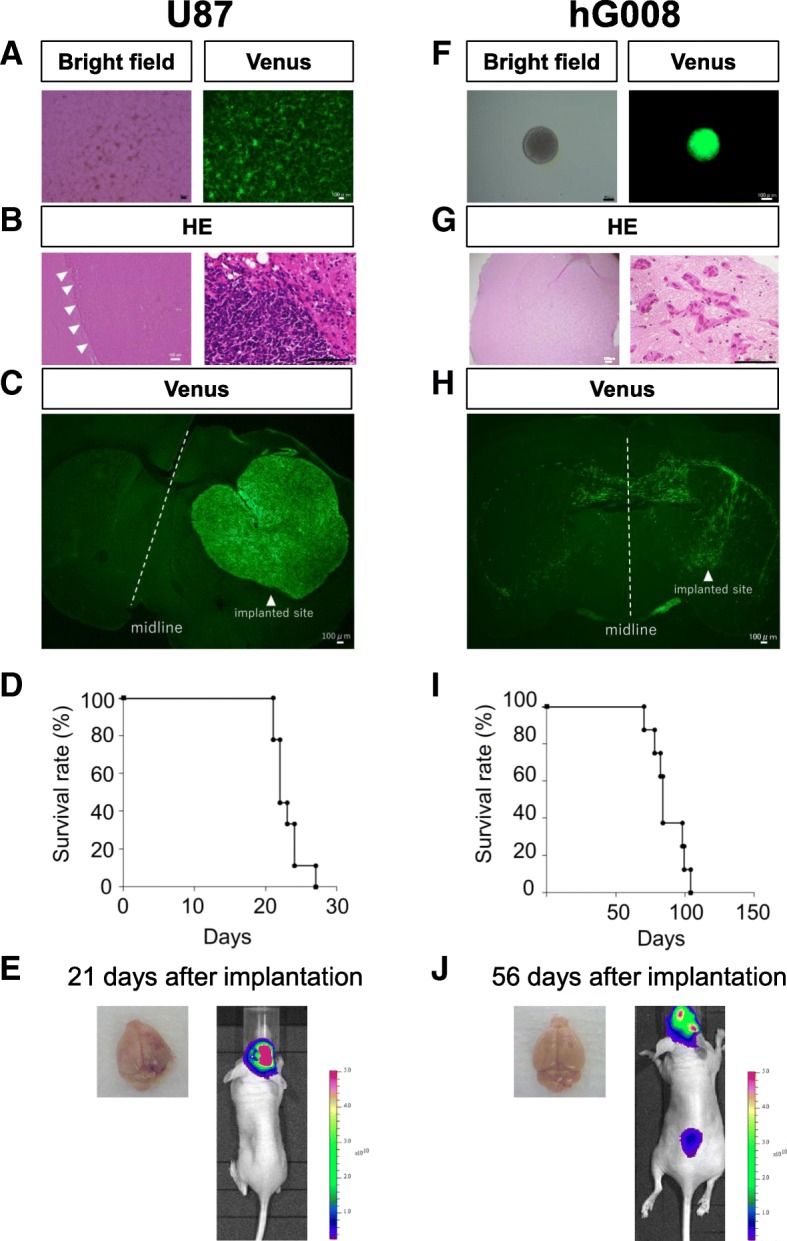
Characteristics of U87 and hG008 cells. **a** and **f** In vitro growth of U87 cells (**a**) and hG008 cells (**f**) expressing *ffLuc*. Scale bar, 100 μm. **b**–**e** and **g**–**j** 1 × 10^5^ U87 cells or hG008 cells were implanted into the striatum of mouse brain. Representative H&E staining (**b** and **g**) and fluorescence images (**c** and **h**) of brain sections at 21 days (**b** and **c**) and 45 days (**g** and **h**) after implantation. Scale bar, 100 μm. **d** and **i** Kaplan-Meier plots showing survival of mice implanted with U87 cells (*n* = 9) (**d**) and hG008 cells (*n* = 8) (**i**). **e** and **j** Representative whole brain pictures and BLI images at 21 days and 56 days after implantation of U87 cells (**e**) and hG008 cells (**j**), respectively. Colored scale bars in (**e**) and (**j**) represent BLI radiance intensity in photons/second/cm^2^/steradian and photons/minute/cm^2^/steradian, respectively

To compare the invasive characteristics, a non-invasive human glioblastoma cell line U87 was used as a negative control. U87 cells expressing *ffLuc* were grown as monolayers in vitro (Fig. 1a). In the brains of mice implanted with 1 × 10^5^ U87 cells in the striatum, a clear delineation of the tumor margin was identified by H&E staining at 21 days after implantation (Fig. 1b). The invasion of U87 cells across the corpus callosum was not observed by Venus fluorescence (Fig. 1c), which was like a metastatic tumor in the human brain. All implanted mice died within 30 days after implantation (Fig. 1d). Tumor was macroscopically observed in the brain (Fig. 1e). Luciferase-based BLI using the IVIS system showed one large peak (Fig. 1e), which was consistent with the fluorescence image of Venus (Fig. 1c).

The human GSC line hG008 expressing *ffLuc* had a sphere-forming capacity (Fig. 1f), but the cell proliferation rate was not different from that of U87 cells. In the brains of mice implanted with 1 × 10^5^ hG008 cells, diffuse invasion with extensive unclear margin was identified by H&E staining at 45 days after implantation. (Fig. 1g). The invasion of hG008 cells across the corpus callosum was observed by Venus fluorescence, resulting in bi-hemispheric lesions that had a winged-like appearance (Fig. 1h). The tumor invasion was similar to the histological features of glioblastoma patients. All implanted mice progressively weakened and died 2–3 months after implantation (Fig. 1i). It was difficult to macroscopically distinguish tumor from normal cells (Fig. 1j). BLI showed two peak signals, which reflected the winged-like appearance (Fig. 1j).

### Time-lapse imaging of organotypic brain slice culture

**Fig. 2:**
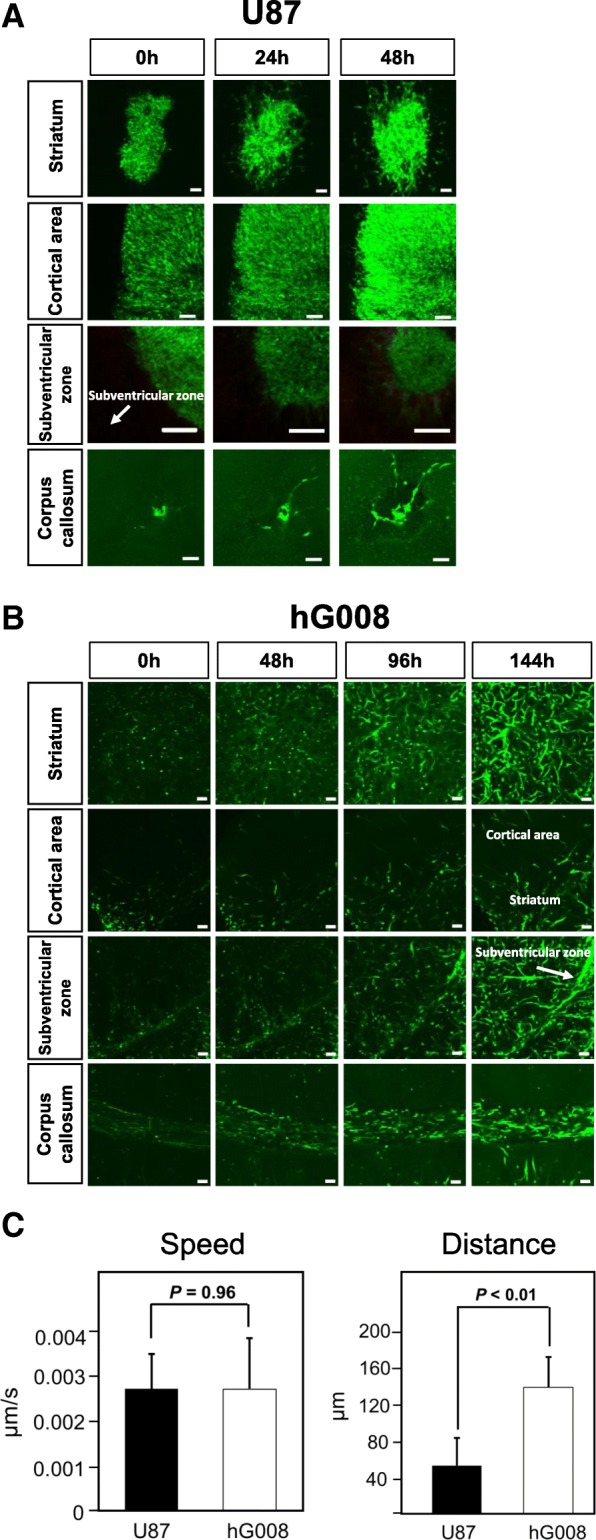
Time-lapse imaging of organotypic brain slice culture. U87 cells were implanted in the right striatum, cortical area, subventricular zone, or corpus callosum. hG008 cells were implanted in the right striatum. Brain tissues from mice 7 days and 45 days after implantation of U87 cells and hG008 cells, respectively, were used for time-lapse imaging of brain slice cultures. See Additional files 2, 3, 4, 5 and 6. **a** and **b** Snapshot fluorescence images of the striatum, cortical area, subventricular zone, and corpus callosum with U87 cells (**a**) and hG008 cells (**b**) at the indicated time points are shown. Scale bar, 100 μm. **c** Quantitative analysis of migration speed and distance of U87 cells and hG008 cells in the striatum. Imaris software was used to track migration of individual cells and calculate the total distance, net distance, and speed of migration. Migration speed was calculated during the period from 48 h to 86 h of culture. Migration distance was measured as the net distance between the initial position after 48 h of culture and the final position after 86 h of culture. Data represent the mean ± SEM (U87, *n* = 10; hG008, *n* = 22)


Since U87 cells form non-invasive tumor mass, U87 cells were implanted in the striatum, cortical area, subventricular zone, or corpus callosum. Time-lapse imaging of organotypic brain slice cultures showed that U87 cells formed a large mass structure in all implanted areas (Fig. 2a, Additional file 2). A few U87 cells invaded from the tumor core. U87 cells implanted in the corpus callosum migrated randomly, and no directional preference was observed (Fig. 2a).

**Fig. 3:**
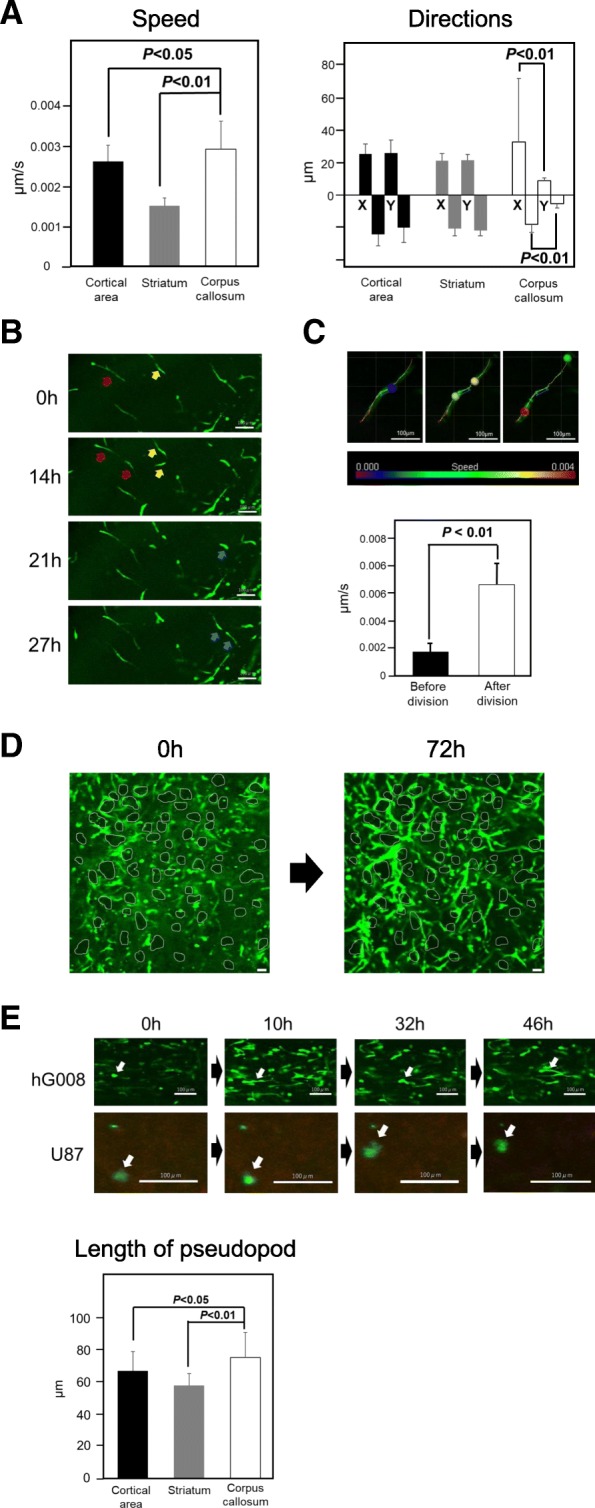
Migration of hG008 cells in different brain areas of slice culture. **a** Speed and direction of hG008 cell migration in the cortical area, striatum, and corpus callosum were analyzed using Imaris software during the period from 48 h to 96 h of culture. Data represent the mean ± SEM (*n* = 56). **b** Snapshot images of hG008 cell division in the striatum and cortical area of contralateral side at the indicated time points are shown. Arrows (red, yellow, and blue) indicate dividing cells. **c** Migration speed was calculated for 12 h before and after cell division using Imaris software. Data represent the mean ± SEM (*n* = 12). Snapshot images of cell division are shown. Colored scale bar represents cell migration speed (μm/second). **d** Migration of hG008 cells along the vertically passing axon bundles in the striatum. Snapshot images after 0 h and 72 h of culture are shown. Areas enclosed by white lines depict axon bundles. Scale bar, 100 μm. **e** The length of pseudopod of hG008 cells was quantified using ImageJ software during the period from 48 h to 96 h of culture. Data represent the mean ± SEM (*n* = 18). Snapshot images of hG008 cells and U87 cells in the corpus callosum at the indicated time points are shown. Scale bar, 100 μm

**Additional file 2:** Time-lapse imaging of organotypic brain slice cultures (Related to Figs. 2 and 3). Time-lapse imaging of the striatum with U87 cells during the first 48 h of culture. Images were captured every 20 min. (MP4 902 kb)


In contrast, time-lapse imaging of slice cultures revealed the highly invasive characteristics of hG008 cells in different brain areas (Additional files 3, 4, 5 and 6). In the striatum, where hG008 cells were implanted, hG008 cells diffusely invaded (Fig. 2b). In the cortical area, hG008 cell density was low for 144 h. Around inferior horn of the lateral ventricle, hG008 cells tended to invade toward the wall of the ventricle. In the corpus callosum, hG008 cells migrated predominantly in one direction with a to-and-fro motion. Once hG008 cells entered into the space of corpus callosum, they rarely exited (Fig. 2b). There was no significant difference in migration speed between U87 cells and hG008 cells, while the distance between the start and end points of hG008 cells was longer than that of U87 cells (Fig. 2c).


**Additional file 3:** Time-lapse imaging of organotypic brain slice cultures (Related to Figs. 2 and 3). Time-lapse imaging of the striatum with hG008 cells during the 144-h culture period. Images were captured every 20 min. (MP4 2203 kb)



**Additional file 4:** Time-lapse imaging of organotypic brain slice cultures (Related to Figs. 2 and 3). Time-lapse imaging of the cortical area with hG008 cells during the 144-h culture period. Images were captured every 20 min. (MP4 2156 kb)



**Additional file 5:** Time-lapse imaging of organotypic brain slice cultures (Related to Figs. 2 and 3). Time-lapse imaging of the subventricular zone with hG008 cells during the 144-h culture period. Images were captured every 20 min. (MP4 2192 kb)



**Additional file 6:** Time-lapse imaging of organotypic brain slice cultures (Related to Figs. 2 and 3). Time-lapse imaging of the corpus callosum with hG008 cells during the 144-h culture period. Images were captured every 20 min. (MP4 2135 kb)


The migration speed of hG008 cells in the corpus callosum was the fastest among three areas analyzed (Fig. 3a). The migration direction of hG008 cells in the corpus callosum exhibited a uniform direction compared with that in other areas (Fig. 3a). During the 144-h observation period, hG008 cells underwent cell division several times in all analyzed areas including the contralateral side. During cell division, the migration temporarily stopped, then two daughter cells migrated in the reverse direction (Fig. 3b). At the moment of cell division, daughter cells rebounded off each other, leading to the fastest movement (Fig. 3c). In the striatum, hG008 cells migrated along axon bundles (Fig. 3d). hG008 cells exhibited longer pseudopods at the moment of migration compared with U87 cells. The length of pseudopod of hG008 cells was the longest in the corpus callosum (Fig. 3e).

### Differentiation of GSCs

**Fig. 4:**
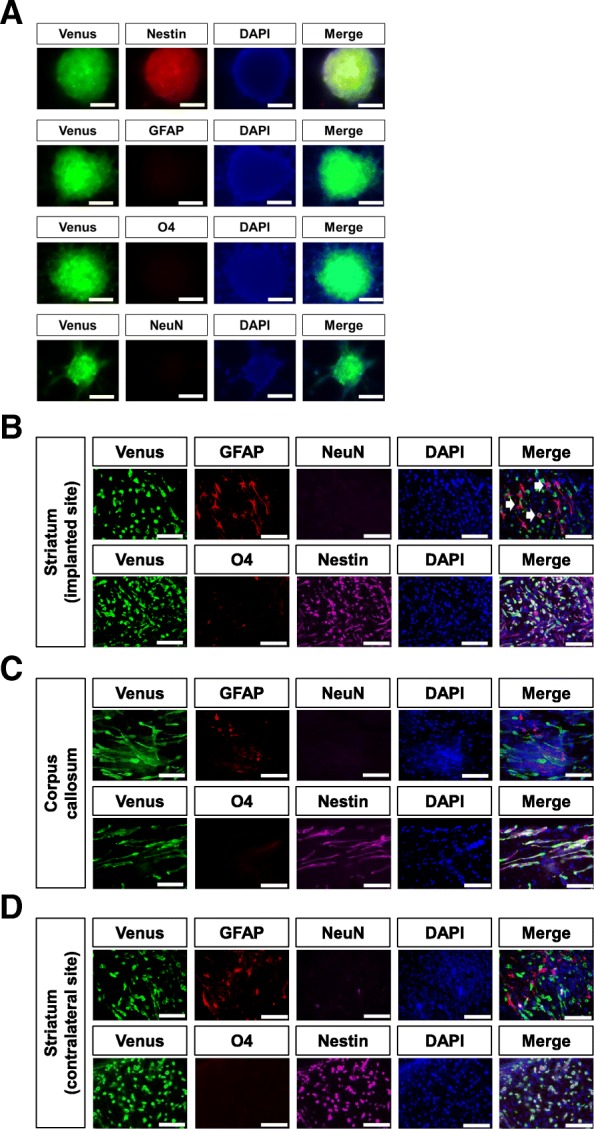
Differentiation of hG008 cells. **a** hG008 cells cultured under serum-free sphere-forming conditions were immunostained with anti-Nestin, anti-GFAP, anti-O4, and anti-NeuN antibodies. Scale bar, 100 μm. **b**–**d** Sections from brain slice cultures (after 96 h of culture) obtained from mice implanted with hG008 cells were immunostained with anti-Nestin, anti-GFAP, anti-O4, and anti-NeuN antibodies. White arrows indicate GFAP-positive hG008 cells. Scale bar, 100 μm

hG008 cells have the ability to differentiate into cells of astroglial, neuronal, and oligodendroglial lineages in vitro [18]. Under the serum-free sphere-forming culture condition, hG008 cells expressed Nestin, a neural stem/progenitor cell marker, but neural differentiation markers, GFAP, O4, and NeuN, were not expressed (Fig. 4a).

Immunostaining of brain slice cultures showed a few GFAP-positive hG008 cells at the implanted striatum, indicating astrocytic differentiation (Fig. 4b). However, no GFAP-positive hG008 cells were observed in other areas (Fig. 4c, d). No hG008 cells expressing O4 or NeuN were detected. Most hG008 cells invading to various arias expressed Nestin (Fig. 4b, c, d), suggesting that hG008 cells retained stemness during migration.

### Directional migration of GSCs

**Fig. 5:**
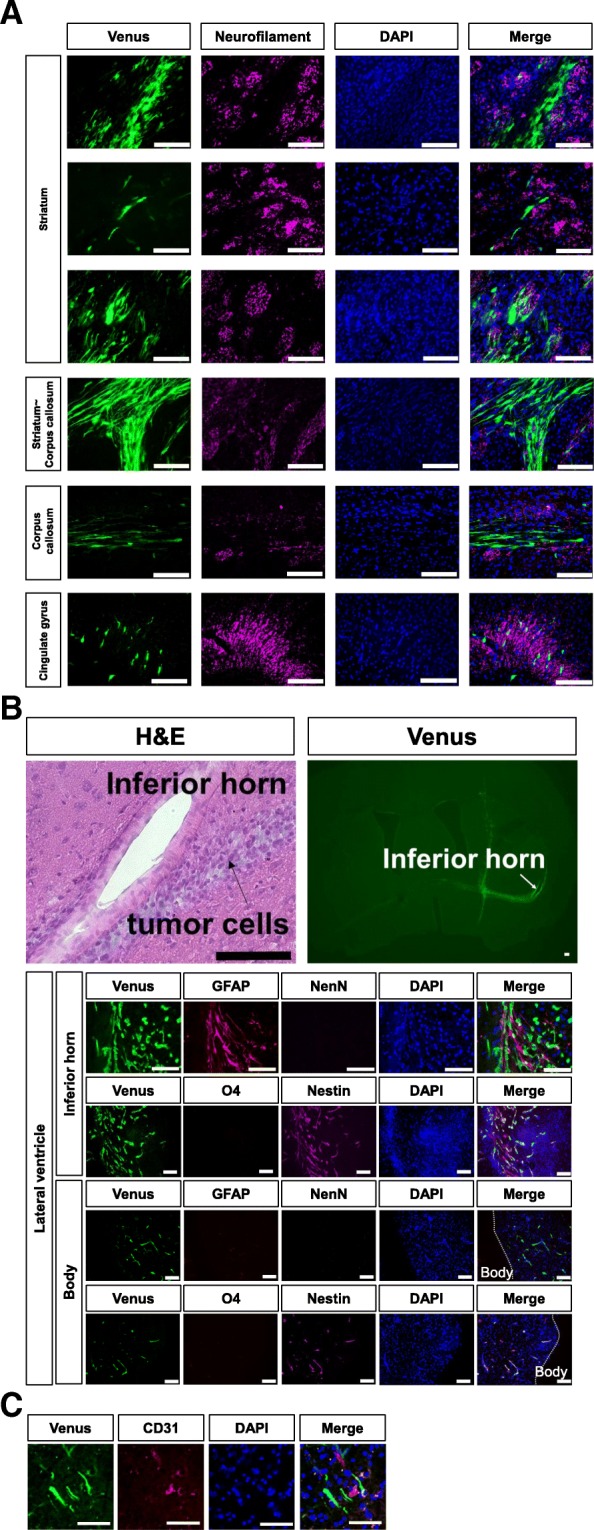
Directional migration of hG008 cells in vivo. Brain sections from mice implanted with hG008 cells were immunostained with anti-Neurofilament protein, anti-Nestin, anti-GFAP, anti-O4, anti-NeuN, and anti-mouse CD31 antibodies. **a** Migration along axonal fibers in the striatum, transitional zone between striatum and corpus callosum, corpus callosum, and cingulate gyrus is observed. Scale bar, 100 μm. **b** H&E staining and fluorescence images are shown. Directional migration toward the inferior horn of the lateral ventricle is observed. Scale bar, 100 μm. **c** Localization in the perivascular area was analyzed by immunostaining for CD31, an endothelial cell marker. Scale bar, 100 μm

Immunohistochemical analysis was performed on brain sections from mice implanted with hG008 cells. hG008 cells migrated along axonal fibers, as visualized by immunostaining for neurofilament protein, in the striatum, corpus callosum, and cingulate gyrus (Fig. 5a). Of note, most hG008 cells in the corpus callosum migrated unidirectionally, and hG008 cells were rarely identified outside the corpus callosum (Fig. 5a). hG008 cells exhibited strong directional migration toward the inferior horn of the lateral ventricle in comparison to the body of the lateral ventricle, and H&E staining demonstrated that hG008 cells were concentrated around the inferior horn (Fig. 5b). Moreover, hG008 cells were observed around GFAP-positive mouse cells attaching the wall of the inferior horn. hG008 cells were often found in the perivascular area of large-sized blood vessels (Fig. 5c).

### 3D imaging of GSCs using whole-brain clearing

**Fig. 6:**
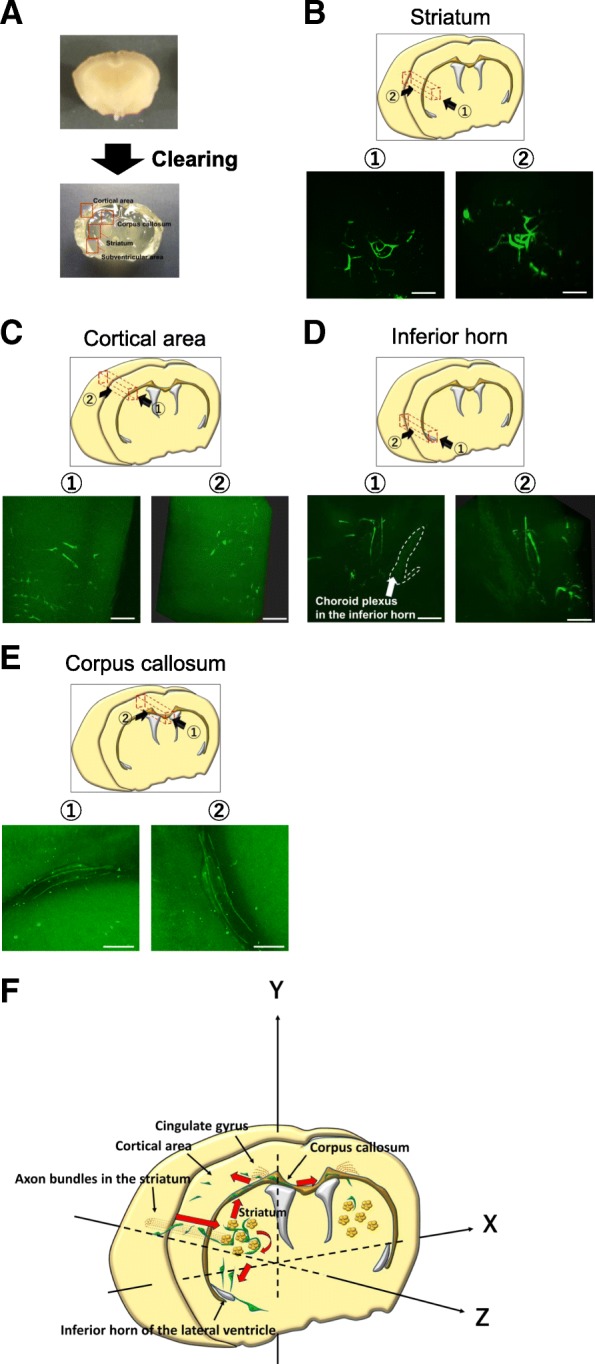
Whole-brain imaging of GSC invasion. Brain tissues from mice 45 days after implantation of hG008 cells were cleared and 3D fluorescence images were reconstructed. See Additional files 7, 8, 9 and 10. **a** Clearing of a 4-mm thick brain slice. **b**–**e** Representative fluorescence images of the striatum (**b**), cortical area (**c**), inferior horn of the lateral ventricle (**d**), and corpus callosum (**e**) are shown. The imaged area and direction are indicated. Scale bar, 100 μm. **f** Schematic illustration of GSC invasion in the brain. GSCs invaded along axon bundles from the implanted site toward the contralateral side through the corpus callosum. GSCs also invaded toward the inferior horn of the lateral ventricle. Red arrows indicate the direction of GSC migration


Brain tissues at 45 days after implantation of hG008 cells were optically cleared, and 3D fluorescence images of the striatum, cortical areas, inferior horn of the lateral ventricle, and corpus callosum were obtained (Fig. 6a, Additional files 7, 8, 9 and 10). 3D images of the striatum revealed that hG008 cells migrated in a helical pattern, twisting around axon bundles. The diameter of helical movement was approximately 100 μm, which was similar to that of axon bundles in the striatum (Fig. 6b). This was consistent with the results of brain slice cultures (Fig. 3d). In the cortical area, tumor cell density was low, and migration along the anteroposterior axis (the Z-axis) was rarely detected (Fig. 6c). 3D images of the subventricular zone of the inferior horn showed migration toward the inferior horn from all directions (Fig. 6d). 3D images of the corpus callosum confirmed unidirectional migration (Fig. 6e). These invasion patterns are summarized in the scheme shown in Fig. 6f.

**Additional file 7:** 3D fluorescence images of optically cleared brains implanted with hG008 cells (Related to Fig. 6). 3D images of the striatum. (WMV 2025 kb)



**Additional file 8:** 3D fluorescence images of optically cleared brains implanted with hG008 cells (Related to Fig. 6). 3D images of the cortical area. (WMV 2087 kb)



**Additional file 9:** 3D fluorescence images of optically cleared brains implanted with hG008 cells (Related to Fig. 6). 3D images of the inferior horn of the lateral ventricle. (WMV 1456 kb)



**Additional file 10:** 3D fluorescence images of optically cleared brains implanted with hG008 cells (Related to Fig. 6). 3D images of the corpus callosum. (WMV 1134 kb)


## Discussion

Although invasion of GSCs in a living organism has been previously reported by using time-lapse imaging of brain slice cultures, only a small area around the implanted site was analyzed [19, 20]. Recently, 3D brain imaging has been obtained using the tissue-clearing method, in which the architecture of neural circuits has been clearly identified [24, 26, 27]. In the present study, using time-lapse imaging of organotypic brain slice cultures and 3D imaging of optically cleared whole brains, the invasion of human hG008 GSCs was spatiotemporally visualized in multiple brain areas.

Non-invasive glioma cells, such as U87 cells, migrate in a fibroblast-like manner, extending a broad lamellipodium [31, 32]. In contrast, GSCs migrate like neural progenitor cells (NPCs) with a mesenchymal mode of motility, in which a leading portion of polarized cell extends forward and movement is a traction-dependent manner [31, 33–35]. The focal adhesions to the extracellular matrix (ECM) that recruit ECM-degrading proteolytic enzymes lead to ECM remodeling and generation of corridors for migration. hG008 GSCs showed the longest pseudopod and rapid migration in the corpus callosum. Before cell division, hG008 cells temporarily squeezed, then daughter cells rebounded off each other with the fastest movement. These observations add new information to the characteristics of GSC migration. As demonstrated in this study, glioma cells are known to reside in perivascular spaces [35]. Invasive glioma cells change the morphology of peritumoral microvessels by inducing intussusceptive microvascular growth and capillary loop formation, which may be beneficial for glioma growth [36].

Glioma cells are known to invade along white matter tracts [35], which are suggested to contain chemoattractants. Neurofilament, a neuronal intermediate filament essential for the radial growth of axons during development, was used to detect axon bundles [37]. GSC migration along axon bundles was shown in the striatum by immunostaining for neurofilament protein. This directional preference was made even clearer by the analysis of Z-axis component obtained by 3D imaging of cleared whole brains. GSCs migrated in a helical pattern around axon bundles, leading to invasion in both the rostral and caudal directions from the implanted area.

Directional migration of GSCs may be associated with the anatomic features. The corpus callosum, composed of callosal axons, is the major commissural tract connecting the cortical regions of the right and left hemispheres. Thin fibers are observed in the anterior corpus callosum (genu), and the large myelinated fibers are preferentially located in the posterior region (splenium) [38, 39]. Since our results demonstrate that GSCs tend to migrate toward the anterior corpus callosum rather than posterior lesion with the fastest moving speed and almost all GSCs do not divagate from the corpus callosum, the axonal density may be associated with the direction and speed of GSC migration. In contrast, GSCs do not tend to migrate toward the cortical area, which may be associated with complex and tight connections of neurons. These preferences in the direction of GCS migration may be related to a “butterfly” appearance of glioblastoma.

GSCs also displayed directional migration toward various directions, including the inferior horn of the lateral ventricle, that were independent of the axonal distributions. NPCs may be involved in the tropism of GSCs, because NPCs in the subventricular zone have been shown to secrete the neurite outgrowth-promoting factor pleiotrophin that is one of key chemoattractants for glioma invasion [40]. Indeed, we observed many GSCs around GFAP-positive mouse cells located in the subventricular zone of the inferior horn but not in the body of the lateral ventricle.

The relationship between invasive migration and differentiation of GSCs has not been fully elucidated [41]. We identified a few GFAP-positive astrocytes differentiated from hG008 GSCs only at the striatum around the implanted site. In contrast, GSCs invading the corpus callosum, striatum, and cortical area of the contralateral side were kept undifferentiated. Furthermore, GSCs at the contralateral side underwent cell division several times, indicating that the invasive GSCs maintained proliferative ability and did not undergo terminal differentiation. These results suggested that differentiation of GSCs after migration takes a certain amount of time, although the brain slice culture is of limited duration (~ 7 days).

Spatiotemporal visualization of GSC migration is informative. Our observations demonstrate various invasive characteristics of human hG008 GSCs, which share histological features observed in glioblastoma patients. Orthotopic xenografts of hG008 GSCs would serve as a good model to study GSC invasion and to evaluate possible therapies. Understanding the mechanisms underlying GSC invasion is essential for the development of effective therapy to inhibit diffuse infiltration. Spatiotemporal visualization techniques can be applied to other patient-derived GSCs in xenograft models and may be useful for patient-specific screening of anti-migration therapeutic agents.

## Additional files

Additional file 1: Magnetic resonance imaging (MRI) of the patient from whom hG008 GSC line was derived. FLuid-Attenuated Inversion Recovery (FLAIR) images demonstrate the invasion into the corpus callosum. T1-weighted images with gadolinium (Gd) contrast enhancement demonstrate ring-enhanced lesion on the parietal lobe, which is typical image of glioblastoma. (PDF 98 kb)

## Abbreviations

3D: Three-dimensional
BLI: Bioluminescence imaging
ECM: Extracellular matrix
EF-1α: Elongation factor 1 α subunit
GSC: Glioma stem cell
NPC: Neural progenitor cell
PBS: Phosphate-buffered saline
PFA: Paraformaldehyde
