# Supplementary figures and images for: Visualization of spatiotemporal dynamics of human glioma stem cell invasion
Source: Mol Brain. 2019 May 6;12:45. doi: 10.1186/s13041-019-0462-3 (PMC6503361; doi:10.1186/s13041-019-0462-3)

**Additional file 1**

**FLAIR**

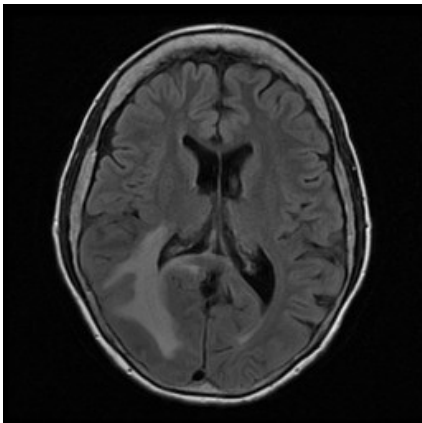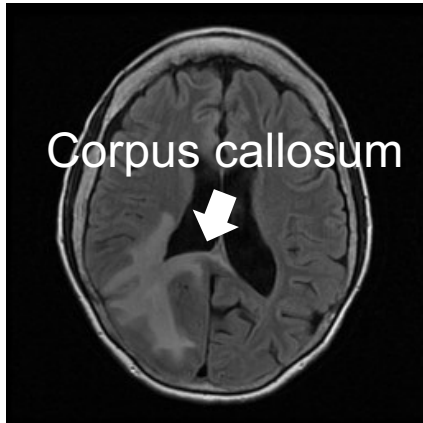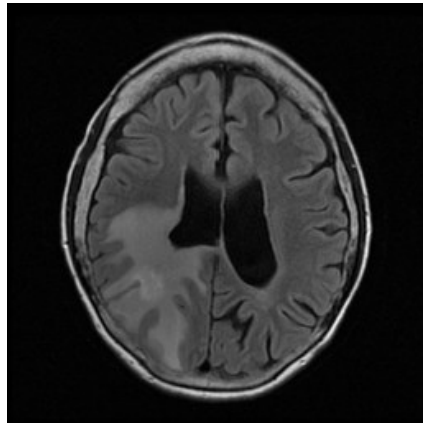

**Gd +**

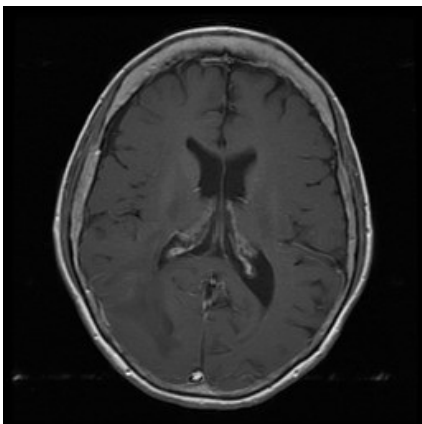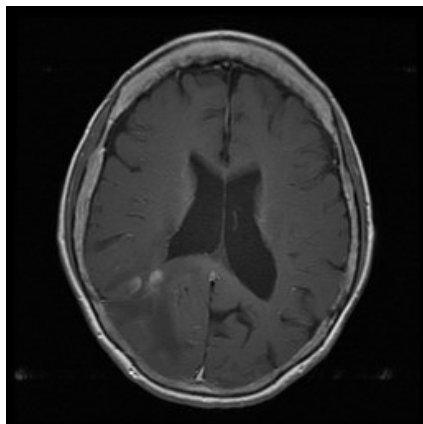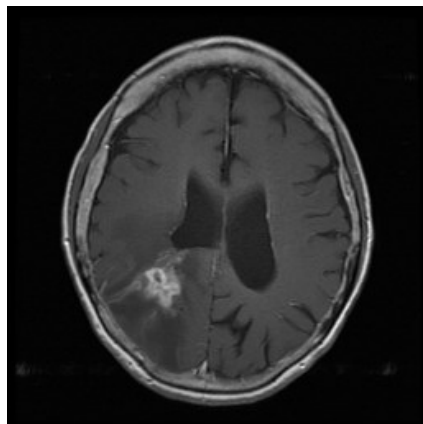

Supplement: Supplementary file 10 — Magnetic resonance imaging (MRI) of the patient from whom hG008 GSC line was derived. FLuid-Attenuated Inversion Recovery (FLAIR) images demonstrate the invasion into the corpus callosum. T1-weighted images with gadolinium (Gd) contrast enhancement demonstrate ring-enhanced lesion on the parietal lobe, which is typical image of glioblastoma. (PDF 98 kb) [file 13041_2019_462_MOESM1_ESM.pdf]
